# Does Thoracodorsal Pedicle Dissection Affect Upper-Extremity Lymphatic Drainage? Arm Circumference After Free Latissimus Dorsi Muscle Flap Harvest in Patients Without Axillary Lymphadenectomy

**DOI:** 10.3390/jcm15187285

**Published:** 2026-09-19

**Authors:** Maximilian C. Stumpfe, Moritz Billner, Celena Sörgel, Thomas Seßler, Chiara Kantlehardt, Denis Ehrl

**Affiliations:** 1 Department of Plastic, Reconstructive and Hand Surgery, Burn Centre for Severe Burn Injuries, Nuremberg Clinics, Paracelsus Medical University, Breslauerstrasse 201, 90471 Nuremberg, Germany; 2 Department of Orthopedics and Trauma Surgery, Musculoskeletal University Center Munich (MUM), LMU University Hospital, LMU Munich, Marchioninistraße 15, 81377 Munich, Germany

**Keywords:** latissimus dorsi flap, donor-site morbidity, lymphoedema, thoracodorsal artery, axillary reverse mapping, free flap, microsurgery

## Abstract

**Background**: The free latissimus dorsi (LD) muscle flap remains a workhorse for large soft-tissue defects. Its pedicle, however, must be followed proximally into the axilla—into the immediate vicinity of the lymphatics that drain the arm—and whether this compromises upper-extremity drainage has never been settled. The few studies that examined lymphoedema after LD surgery relied on questionnaires and were confined to breast reconstruction, where axillary clearance and irradiation offer a sufficient explanation for anything they found; the harvest itself has never been isolated. **Methods**: We identified every patient who underwent free LD muscle flap reconstruction for defect coverage at our institution between September 2017 and December 2022 and examined those who attended a standardized cross-sectional follow-up. None had undergone axillary lymph node dissection, sentinel node biopsy, or axillary irradiation, and none had received cervical or supraclavicular irradiation ipsilateral to the donor side. A single examiner measured upper-arm and forearm circumference bilaterally, 10 cm proximal and 10 cm distal to the medial epicondyle. Patients whose flap had been transferred to an upper limb were excluded, because in them the tape measures the flap rather than the donor site. The contralateral limb served as the intra-individual control. The primary endpoint was the mean side difference in upper-arm circumference with its 95% confidence interval. **Results**: Forty-one patients (25 women, 16 men; mean age 54.4 ± 16.5 years; mean follow-up 14.4 ± 12.0 months, range 3–48) were included. Upper-arm circumference averaged 27.88 ± 3.26 cm on the donor side and 27.77 ± 3.65 cm contralaterally—a difference of +0.11 cm (95% CI −0.28 to +0.50; *p* = 0.57). Forearm values were 23.82 ± 2.89 cm and 23.94 ± 3.07 cm (−0.12 cm; 95% CI −0.47 to +0.23; *p* = 0.49). Two of 41 patients (4.9%; exact 95% CI 0.6–16.5%) crossed the conventional 2 cm threshold at the upper arm—and exactly as many crossed it in the opposite direction, with the donor limb the smaller of the two. Side difference bore no relation to time since surgery. **Conclusions**: Free LD muscle flap harvest showed no detectable difference in arm circumference at two standardized levels in patients who had never undergone axillary lymphadenectomy. The upper-arm mean difference is compatible with no more than a 0.5 cm increase, and threshold crossings occurred equally often in both directions. Circumference is a coarse surrogate that cannot detect subclinical (International Society of Lymphology [ISL] stage 0) lymphoedema; within that limit, these data give no support for avoiding the flap on lymphatic grounds.

## 1. Introduction

Free tissue transfer remains the mainstay of reconstruction for extensive soft-tissue defects, whether these follow trauma, tumor resection, infection, or chronic wound breakdown [1,2]. Among available donors, the free latissimus dorsi (LD) muscle flap has held its ground for four decades, owing to its size, consistent anatomy, long pedicle, low donor-site morbidity, and adaptability across anatomical regions [2,3,4].

The latissimus dorsi is the largest flat muscle in the body. It arises from the thoracolumbar fascia, the lower thoracic spinous processes, the iliac crest, the lower ribs, and the inferior angle of the scapula and inserts near the crest of the lesser tubercle of the humerus. Together with teres major, it forms the posterior axillary fold—an intimacy with the axilla that matters here. The thoracodorsal nerve innervates it; the thoracodorsal artery, a branch of the subscapular artery, supplies it [5]. Mathes and Nahai classified it as Type V: one dominant pedicle, multiple secondary segmental perforators [6].

That anatomy is the source of the concern. Secondary upper-extremity lymphoedema arises when axillary lymphatic drainage is disrupted, as it is by axillary lymph node dissection (ALND) and regional nodal irradiation [7,8,9]. Axillary reverse mapping (ARM) has shown that the nodes and lymphatics draining the arm sit beneath the axillary vein and lateral to the thoracodorsal bundle, hard against the latissimus dorsi [10,11]. The dissection corridor, in other words, is not remote from the arm’s lymphatic pathway but immediately alongside it. The worry that harvest—and the scarring that follows it—might impair lymphatic transport in the ipsilateral limb is therefore anatomically plausible, not fanciful.

The literature does not resolve it. Lymphoedema has occasionally appeared as an outcome in LD donor-site studies, but always by patient self-report and always in breast reconstruction [12]. There, prior axillary clearance and irradiation account for whatever is observed, and the contribution of the harvest cannot be teased out. We are aware of no objective measurement of the arm after free LD harvest in patients who never underwent axillary nodal surgery. We therefore studied a cohort in which the dominant confounder is absent by design.

## 2. Materials and Methods

### 2.1. Study Design and Reporting

We combined retrospective identification of a consecutive surgical cohort with a prospective, standardized cross-sectional examination at a single center. Reporting follows the STROBE statement for observational studies. The study was conducted according to the guidelines of the Declaration of Helsinki and approved by the Institutional Ethics Committee (21-0475, LMU Munich, Munich, Germany). All patients were operated on and examined at the Division of Hand, Plastic and Aesthetic Surgery, LMU University Hospital, Munich. All participants were adults (≥18 years) and gave written informed consent.

### 2.2. Participants

Every patient who underwent free LD muscle flap reconstruction for defect coverage between September 2017 and December 2022 was identified from the institutional database; one plastic surgeon performed all operations. Of 87 patients identified, 15 had died, 6 could not be contacted, and 20 declined participation (long travel distance, acute illness, reduced mobility, or lack of time), leaving 46 who attended, all at least three months after surgery. We also reviewed the electronic records of all 41 patients who did not attend for documented arm swelling or side-to-side circumference asymmetry, a clinical diagnosis of upper limb lymphoedema, prescription of compression garments, complex decongestive therapy, or lymphological referral.

Five patients had received their flap on an upper limb (forearm *n* = 2, hand/forearm *n* = 2, upper arm *n* = 1). In these, arm circumference measures the transferred muscle rather than donor-site morbidity, and they were excluded. This exclusion, and the concordant whole-limb endpoint defined below, were specified before the paired analyses were run, and are described here as pre-planned rather than formally pre-registered. The primary analysis set comprises 41 patients.

We reviewed the chart of every patient in the analysis set specifically for this study. None had undergone axillary lymph node dissection, sentinel lymph node biopsy, or any other axillary nodal surgery; none had received axillary or chest-wall irradiation at any point. For patients treated for head or neck defects, we also confirmed the absence of cervical or supraclavicular radiotherapy ipsilateral to the donor side, since irradiation at the venous angle could impair the arm’s common outflow independently of the axilla. This was the prerequisite for the study question, and it was verified rather than assumed.

### 2.3. Operative Technique

All flaps were harvested by a single senior surgeon using a standardized technique. In every case, the thoracodorsal pedicle was dissected proximally as far as the origin of the subscapular artery, which represents the maximum proximal extent achievable in latissimus dorsi harvest. The vessels were skeletonized within the thoracodorsal corridor; no axillary lymph node tissue was excised, and no clearance of the axillary fat pad was performed. Individual pedicle length was not recorded in the operative reports.

### 2.4. Circumference Measurement

**Figure 1:**
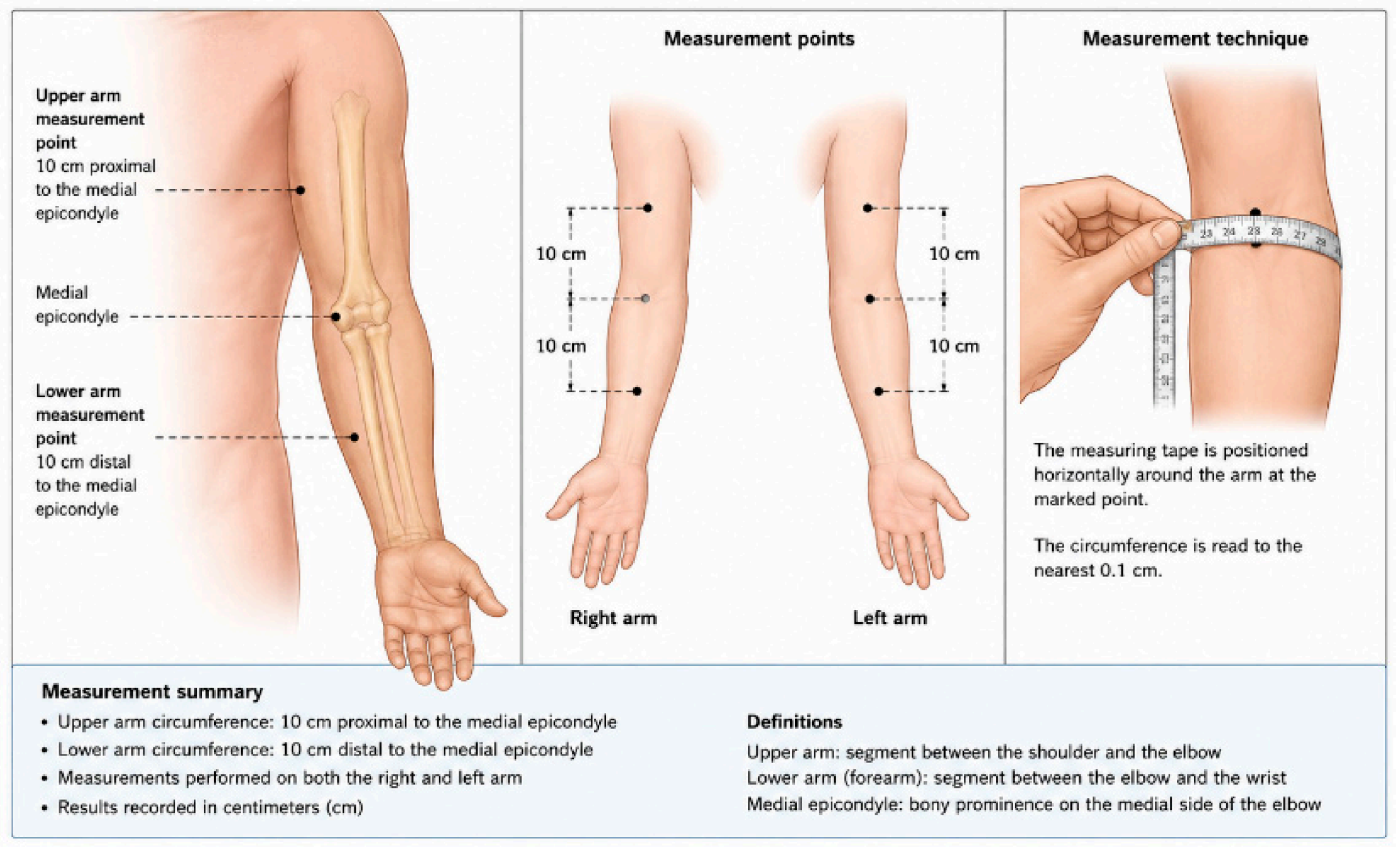
Measurement points for upper-extremity circumference assessment. Circumference measurements were performed bilaterally. The medial epicondyle served as the anatomical reference point. Upper-arm circumference was measured 10 cm proximal to the medial epicondyle, and forearm circumference was measured 10 cm distal to the medial epicondyle; Biorender, 2026.

A single examiner, not the operating surgeon, measured both limbs with a tape measure (Seca GmbH, Hamburg, Germany). The medial epicondyle served as the anatomical reference. Skin marks were placed 10 cm proximal (upper arm) and 10 cm distal (forearm) to the epicondyle on each side, and circumferences were recorded to the nearest 0.5 cm (Figure 1).

The two limbs were measured in sequence by the same examiner, who was not blinded to the donor-side value when recording the contralateral limb; the implications of this for the exact-zero differences are considered in the discussion.

### 2.5. Endpoints

The primary endpoint was the mean side difference in upper-arm circumference (donor minus contralateral) with its 95% confidence interval. Secondary endpoints were the corresponding forearm difference and the proportion of patients whose donor limb exceeded the contralateral by ≥2 cm, the conventional clinical threshold for limb lymphoedema [13]; the proportion in whom the donor limb fell ≥2 cm below the contralateral, used as an internal estimate of physiological asymmetry and measurement noise; and a concordant whole-limb pattern, pre-planned as a donor-side excess of ≥2 cm at both the upper arm and the forearm—the pattern true lymphoedema would produce, as distinct from an isolated single-level deviation.

### 2.6. Statistical Analysis

Paired comparisons used the paired t-test, with the Wilcoxon signed-rank test as a sensitivity analysis. We report mean differences with 95% confidence intervals and give this precedence over *p*-values, because the question is not whether a difference can be detected but whether a clinically relevant one can be excluded. Proportions are reported with exact (Clopper–Pearson) 95% confidence intervals. An exact sign test assessed the symmetry of threshold crossings. Spearman correlation and stratification at 12 months assessed the influence of follow-up duration. Given the small number of events, no multivariable model was fitted, as it would be overfitted. Analyses used Python 3.12 (SciPy, statsmodels); two-sided *p* < 0.05 was considered significant.

## 3. Results

### 3.1. Cohort

Forty-six patients attended follow-up; five were excluded for an upper-limb recipient site, leaving 41 in the primary analysis set (Table 1). Mean follow-up was 14.4 ± 12.0 months (median 10; range 3–48), and 18 patients (43.9%) were seen at least a year after surgery. In the records of the patients who did not attend the examination (15 deceased, 20 who declined participation, 6 not contactable), no arm swelling, no diagnosis of upper-limb lymphoedema, and no lymphoedema-directed treatment were documented. This documentation extended to the completion of wound healing, approximately four weeks after discharge; no further presentation to our institution was documented thereafter.

### 3.2. Handedness and Side Asymmetry

Handedness allowed the one apparent side asymmetry to be resolved. Harvest on the left produced a mean upper-arm difference of +0.25 cm and harvest on the right −0.05 cm—an artifact of dominance, not of side. Only 36% of left-sided harvests were on the dominant limb, against 89% of right-sided ones. Stratified by dominance, the donor-minus-contralateral upper-arm difference was +0.26 ± 1.42 cm when the flap was taken from the dominant side and −0.12 ± 0.87 cm when taken from the non-dominant side (Mann–Whitney *p* = 0.23). At the forearm the two strata did differ (dominant +0.12 ± 1.21 cm, non-dominant −0.50 ± 0.86 cm; *p* = 0.007), but in the direction of a relatively smaller non-dominant donor forearm—the expected footprint of limb dominance, not of lymphatic obstruction. Anchoring the comparison to handedness rather than to harvest side gave the same picture: the dominant limb exceeded the non-dominant by +0.21 cm at the upper arm (95% CI −0.18 to +0.59) and +0.27 cm at the forearm (95% CI −0.08 to +0.61).

### 3.3. Primary Endpoint

Neither level showed a side difference (Table 2). At the upper arm, the donor limb measured 27.88 ± 3.26 cm against 27.77 ± 3.65 cm contralaterally—a difference of +0.11 cm (95% CI −0.28 to +0.50; paired t *p* = 0.57; Wilcoxon *p* = 0.58). At the forearm, 23.82 ± 2.89 cm against 23.94 ± 3.07 cm—a difference of −0.12 cm (95% CI −0.47 to +0.23; paired t *p* = 0.49; Wilcoxon *p* = 0.27). The median side difference was 0.0 cm at both levels.

### 3.4. Threshold Analysis

**Figure 2:**
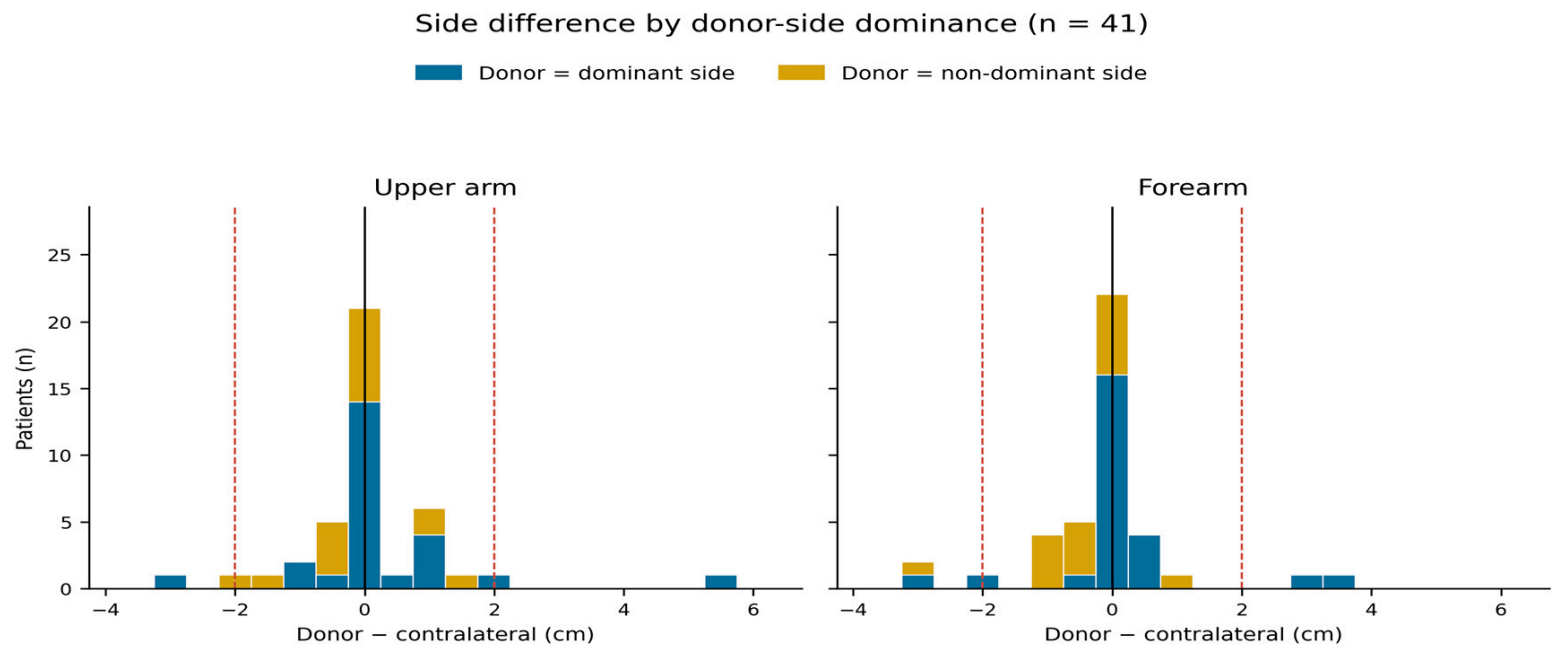
Side differences (donor minus contralateral) in the primary analysis set (n = 41), stacked by whether the flap was harvested from the dominant or non-dominant side. Dashed lines mark the ±2 cm thresholds. The distribution is centered on zero and approximately symmetric, and crossings of either threshold arise from both dominance strata.

Two of 41 patients (4.9%; exact 95% CI 0.6–16.5%) crossed the ≥2 cm threshold on the donor side at the upper arm, and two of 41 (4.9%) at the forearm (Table 3). Crucially, as many or more crossed the threshold the other way—the donor limb smaller than the contralateral (upper arm 2/41, 4.9%; forearm 3/41, 7.3%). Crossings therefore fell in both directions with similar frequency (Figure 2), consistent with physiological asymmetry and measurement variability rather than a directional process. With only two - three events per side, the exact sign test is uninformative (*p* = 1.00 at both levels) and is reported for completeness, not as evidence of symmetry.

A concordant whole-limb pattern—a donor-side excess of ≥2 cm at both levels—occurred in one patient (2.4%; exact 95% CI 0.1–12.9%). Driven by the single event, this interval is wide: the data are compatible with a concordant-pattern rate of up to roughly 13%, and we are careful not to present a null result as more precise than it is. The patient in question underwent head reconstruction, was harvested on the left, measured +5.5 cm at the upper arm and +3.0 cm at the forearm, and was examined at eight months. Chart review found no documented swelling, no compression therapy, no erysipelas, and no venous thrombosis. The finding remains an unexplained outlier and is reported as such.

### 3.5. Follow-Up Duration

Time since surgery had no bearing on side difference (upper arm: Spearman ρ = +0.06, *p* = 0.70; forearm: ρ = −0.31, *p* = 0.05—if anything, the donor forearm grew relatively smaller with time). Patients seen at ≥12 months differed no more than those seen earlier (upper arm −0.08 ± 1.14 cm versus +0.26 ± 1.30 cm). Within the observed window, nothing suggests a late-onset process.

## 4. Discussion

Free LD muscle flap harvest, performed without axillary nodal surgery or irradiation, left no measurable trace on the ipsilateral arm. The mean upper-arm difference was +0.11 cm, and its confidence interval excludes any increase that would matter clinically. Threshold crossings went one way as often as the other—what tape-measure variability and physiological asymmetry produce, and not what lymphoedema produces.

Lymphoedema has been recorded after LD surgery before, but only by questionnaire and only in breast reconstruction, where axillary clearance and irradiation supply a complete explanation for anything observed [12]. That literature even hints that LD transfer may improve pre-existing lymphoedema [12,14,15,16]—a claim we take up below. In none of these settings can the harvest be separated from the axillary surgery that preceded it [17]. Restricting the cohort to head, trunk, and lower-limb reconstruction in patients who never underwent axillary nodal surgery removes the confounder by construction.

Whether the result is biologically coherent turns on where the arm’s lymph actually goes. Cadaveric microdissection shows that dye injected into the upper limb reaches the lateral (humeral) axillary group, which serves the limb exclusively; the breast and posterior thoracic wall drain instead through the anterior (pectoral) and posterior (subscapular) groups [18,19]. ARM has since pinned these arm-draining nodes down in the living patient: most lie beneath the axillary vein and lateral to the thoracodorsal bundle, hard against the latissimus dorsi [10,11].

A further consideration concerns not the lymphatic pathway itself but the blood supply of the nodes along it. Because pedicle dissection proceeds close to the level I axillary nodes, one might ask whether the extensive preparation in this region could, through adhesions or devascularization, alter nodal integrity. The arterial supply of the axillary nodes is, however, both segmental and redundant: cadaveric work shows that the level Ib (mammary sentinel) nodes are fed in a regular paired relationship by the lateral thoracic, thoracodorsal, inferior pectoral, and superficial thoracic arteries, the specific contribution shifting with the branching pattern of the axillary artery trunk [20]. Skeletonizing a single named pedicle—the thoracodorsal vessels—therefore does not remove the collateral arterial network that sustains the surrounding nodes, which is consistent with the absence of any measurable lymphatic consequence in our cohort. This remains an inference from anatomy rather than a demonstration, since we did not image nodal perfusion.

That proximity raises the question, and it is worth resisting the temptation to claim more anatomical distance than exists. The thoracodorsal corridor runs alongside the arm’s lymphatic pathway, not away from it, and ARM lymphatics have been described as lying within the standard axillary dissection field [10]. What separates harvest from lymphadenectomy is not distance but intent. Harvest follows the vessel; lymphadenectomy clears the basin. Free LD harvest excises no nodes, skeletonizes no axillary vein, and never enters the level II and level III compartments medial and posterior to pectoralis minor—the maneuvers that account for the lymphatic cost of ALND. The arm-draining tissue lateral to the thoracodorsal bundle stays where it is. Our data suggest that leaving it there is enough to avoid measurable limb enlargement—though, having measured girth rather than lymphatic transport, we cannot claim to have shown preserved drainage directly. Because dissection extended in every case to the origin of the subscapular artery, the corridor was carried to its maximum proximal extent, so these data address the question at its upper bound rather than at a favorable minimum.

The contrast with oncological axillary surgery sharpens the point. ALND raises the incidence of upper-extremity lymphoedema substantially and combining it with regional nodal irradiation raises it further [7]; reviews of post-mastectomy lymphoedema converge on nodal-basin disruption and radiotherapy as the dominant risks [8,9]. Set against that, the flat circumference signal in this cohort—across a follow-up reaching 48 months—suggests that dissecting a pedicle is not the same insult as clearing a basin.

If LD transfer improves rather than worsens arm lymphoedema, as the breast literature suggests [14,15,16], then a null result here might seem unremarkable. The proposed mechanism shows why the inference does not carry. Improvement after pedicled reconstruction is attributed to releasing axillary scar and importing healthy, non-irradiated, well-vascularized muscle into a fibrotic basin—obliterating dead space and allowing lympho-lymphatic bridging across the flap [14,15]. The mechanism is proposed, not established: the evidence is case reports and small series, and the single prospective study reporting a fall in self-reported lymphoedema was neither designed nor powered for that endpoint [12]. More to the point, it cannot operate in free harvest at all.

Our patients’ muscle left the axilla altogether, transferred to the head, trunk, or lower limb. The axilla was dissected and closed. Nothing vascularized was imported, no scar was released, and dead space was created rather than filled. This cohort met the injurious half of the procedure—pedicle dissection in the axillary fat pad, scarring, seroma—without the therapeutic half. That nothing measurable followed strengthens the inference rather than weakening it. The muscle itself raises a second question. Removing the largest flat muscle in the body might be expected to shift shoulder biomechanics, producing either disuse atrophy or compensatory hypertrophy; we saw neither. Bilateral loading appears to rebalance in the medium term—and limb volume, evidently, is no surrogate for donor-site function after LD harvest [4].

The measurement is the first and largest limitation. Circumference is a coarse instrument: one point per segment, read to the nearest 0.5 cm, cannot resolve subclinical fluid shifts, fat redistribution, or small differences in muscle volume, and two points do not permit truncated-cone volumetry. Perometry, bioimpedance spectroscopy, indocyanine-green lymphography, or MRI volumetry would be needed for that. Twenty-one of 41 patients recorded an upper-arm difference of exactly 0.0 cm; two mechanisms could produce this—digit preference, or an anchoring effect, the latter a real possibility because the examiner was not blinded to the donor-side value when measuring the contralateral limb. Both would blunt small real differences, but neither can manufacture the roughly equal spread of the large crossings in both directions, on which the qualitative conclusion rests. Circumference remains, in any case, blind to subclinical International Society of Lymphology (ISL) stage 0 lymphoedema, in which transport is impaired before girth changes, and a median follow-up of 10 months may be too short for later-onset disease to surface. Secondary lymphoedema of the upper limb may become clinically apparent considerably later, so delayed onset in individual patients cannot be excluded; these results should be read as evidence against an early, harvest-related lymphoedema rather than as evidence of lifelong absence of risk.

We compared against the contralateral limb rather than a preoperative baseline, so pre-existing asymmetry cannot be excluded outright. Its most obvious source, however, could be examined directly: handedness shapes upper-limb asymmetry, and dominant-side strength exceeded non-dominant-side strength in this same cohort. Stratifying the circumference data by dominance left the upper-arm result unchanged and produced only the expected dominance footprint at the forearm (see Results), so handedness does not account for—nor conceal—the null finding. In addition, according to the documented preoperative clinical history, none of the included patients had a history of limb swelling or clinically evident lymphoedema.

Other limitations follow. Without inter- or intra-rater data, measurement precision cannot be quantified. A total of 41 of 87 identified patients did not attend, so selection bias cannot be excluded—though a review of their records documented no arm swelling and no lymphoedema-directed treatment up to the completion of wound healing, and it is unclear why a patient with a swollen donor arm would preferentially decline examination. No validated patient-reported lymphoedema instrument (e.g., LYMQOL, Lymph-ICF) was administered, and body mass index—a major risk factor for secondary lymphoedema—was not recorded. The cohort was nevertheless not a healthy elective population: reconstruction was performed predominantly for wound-healing disorder or infection (46.3%), tumor (31.7%), or trauma (22.0%), and 15 of the 87 patients originally identified had died before recruitment, so the comorbidity burden of this population argues against the null finding being an artifact of unusually low baseline risk. Against these stand a landmark-based protocol applied by one examiner, an intra-individual control, a pre-planned exclusion of measurements contaminated by the flap, a cohort that is large for this particular question, and a population in which the dominant confounder is absent by design. The findings apply to free, muscle-only LD harvest in patients without axillary surgery; they should not be extrapolated to myocutaneous harvest or to the breast-reconstruction setting. Because the pedicle was routinely dissected to the origin of the subscapular artery, a pedicled transfer would not require a more proximal dissection than was performed here; any difference in lymphatic risk between the two techniques is therefore more likely to arise from the muscle remaining within the axilla, from tunneling, and from the myocutaneous design commonly used, than from pedicle length as such. As no pedicled flap was examined, we do not extend our conclusions to that setting.

## 5. Conclusions

In patients who have never undergone axillary lymphadenectomy or irradiation, free latissimus dorsi muscle flap harvest showed no detectable difference in arm circumference at two standardized levels. Threshold crossings occurred with equal frequency in both directions—the pattern of physiological asymmetry rather than lymphoedema—and the mean difference is compatible with no more than a 0.5 cm increase. The thoracodorsal corridor runs close to the arm’s lymphatics, but sparing the nodal basin—whose segmental, redundant arterial supply is not abolished by skeletonizing a single pedicle—appears sufficient to preserve drainage on the evidence available. Circumference cannot capture subclinical disease, so ISL stage 0 lymphoedema cannot be excluded; within that limit, these data give no support for avoiding the flap on lymphatic grounds. Confirmation with volumetric or lymphographic techniques, ideally against a preoperative baseline, is the necessary next step.

## Figures and Tables

**Table 1:** Baseline characteristics of the primary analysis set (*n* = 41).

Characteristic	Value
Age, years—mean ± SD (range)	54.4 ± 16.5 (18–82)
Female/male	25 (61.0%)/16 (39.0%)
Body height, cm—mean ± SD	170.9 ± 9.2
Indication—wound-healing disorder/infection	19 (46.3%)
Indication—tumor	13 (31.7%)
Indication—trauma	9 (22.0%)
Defect site—head	16 (39.0%)
Defect site—lower extremity	19 (46.3%)
Defect site—trunk	6 (14.6%)
Donor side—left/right	22 (53.7%)/19 (46.3%)
Follow-up, months—mean ± SD (range)	14.4 ± 12.0 (3–48)
Follow-up ≥ 12 months	18 (43.9%)
Handedness—right/left	35 (76.1%)/11 (23.9%) [full cohort]
Donor side dominant/non-dominant	25 (61.0%)/16 (39.0%)

**Table 2:** Donor limb versus contralateral limb (*n* = 41); mean ± SD. *p* from paired t-test; Wilcoxon signed-rank *p* = 0.58 (upper arm), 0.27 (forearm).

Segment	Donor (cm)	Contralateral (cm)	Mean Difference (95% CI)	*p*
Upper arm	27.88 ± 3.26	27.77 ± 3.65	+0.11 (−0.28 to +0.50)	0.57
Forearm	23.82 ± 2.89	23.94 ± 3.07	−0.12 (−0.47 to +0.23)	0.49

**Table 3:** Threshold crossings in both directions (*n* = 41).

Segment	Donor ≥ +2 cm (95% CI)	Donor ≤ −2 cm	Sign Test *p*
Upper arm	2 (4.9%; 0.6–16.5)	2 (4.9%)	1.00
Forearm	2 (4.9%; 0.6–16.5)	3 (7.3%)	1.00
Both levels ≥ +2 cm	1 (2.4%; 0.1–12.9)	—	—

## Data Availability

The de-identified data supporting the reported results are available from the corresponding author on reasonable request.
